# Interplays between Soil-Borne Plant Viruses and RNA Silencing-Mediated Antiviral Defense in Roots

**DOI:** 10.3389/fmicb.2016.01458

**Published:** 2016-09-15

**Authors:** Ida Bagus Andika, Hideki Kondo, Liying Sun

**Affiliations:** ^1^State Key Laboratory of Crop Stress Biology for Arid Areas, College of Plant Protection, Northwest A&F UniversityYangling, China; ^2^Group of Plant-Microbe Interactions, Institute of Plant Science and Resources, Okayama UniversityKurashiki, Japan

**Keywords:** soil-borne virus, RNA silencing, antiviral defense, roots, silencing suppressor, *Polymyxa*, *Olpidium*, nematode

## Abstract

Although the majority of plant viruses are transmitted by arthropod vectors and invade the host plants through the aerial parts, there is a considerable number of plant viruses that infect roots via soil-inhabiting vectors such as plasmodiophorids, chytrids, and nematodes. These soil-borne viruses belong to diverse families, and many of them cause serious diseases in major crop plants. Thus, roots are important organs for the life cycle of many viruses. Compared to shoots, roots have a distinct metabolism and particular physiological characteristics due to the differences in development, cell composition, gene expression patterns, and surrounding environmental conditions. RNA silencing is an important innate defense mechanism to combat virus infection in plants, but the specific information on the activities and molecular mechanism of RNA silencing-mediated viral defense in root tissue is still limited. In this review, we summarize and discuss the current knowledge regarding RNA silencing aspects of the interactions between soil-borne viruses and host plants. Overall, research evidence suggests that soil-borne viruses have evolved to adapt to the distinct mechanism of antiviral RNA silencing in roots.

## Introduction

Most plant virus transmissions in nature are facilitated by biological vectors, and the site of virus entry into the host plant differs according to these transmission vectors (Hull, 2013). The majority of plant viruses are transmitted into the aerial plant parts by a variety of arthropods, mainly sap-sucking insects such as aphids and whiteflies, while some soil-inhabiting zoosporic organisms and root-feeding nematodes transmit a number of plant viruses into roots (Hull, 2013). Thus, compatibility of the virus with the tissue or cell where it initially enters the host plant is critical for establishing the infection. Each plant organ or tissue has a distinct metabolism and pronounced physiological characteristics. In particular, the features of plant shoots and roots largely diverged from one another; they differ in their anatomical structures, cell compositions, gene expression patterns, and are exposed to contrasting environmental conditions between above and below ground environments. Consequently, antiviral defense in roots may operate differently than that in shoots, and viruses may have evolved to adapt to these mechanistic differences.

Soil-borne viral diseases are generally difficult to control with conventional chemical or agronomical methods because viruliferous vectors could be widespread underground. In particular, viruliferous resting spores of the zoosporic vectors could be stable and persistent in the infested soil for decades (Rochon et al., 2004; Bragard et al., 2013; Tamada and Kondo, 2013). Consequently, the disease control-measures are mainly dependent on natural plant resistance resources (Kanyuka et al., 2003; Kühne, 2009; McGrann et al., 2009; Ordon et al., 2009), but in agricultural systems, the emergence of resistance-breaking viruses poses a serious threat to crop production (Kühne, 2009; Tamada and Kondo, 2013; Tamada et al., 2016). Nevertheless, studies about the mechanisms by which the plant antiviral defense system combats viruses entering the roots are scarce. This is partly due to the fact that only a limited number of plant-virus–soil-inhabiting vector inoculation systems has been so far successfully established under laboratory conditions.

RNA silencing is a general term for down-regulation of gene expression, mediated by small RNAs in eukaryotes (Baulcombe, 2005). In the cell, RNA silencing is involved in diverse biological processes and operates by targeting DNA/RNA of endogenous or exogenous origin in a nucleic acid sequence-specific manner via inhibition of RNA transcription (involving RNA-directed DNA methylation, RdDM), cleavage of RNA, or translational inhibition of mRNA (Ghildiyal and Zamore, 2009; Voinnet, 2009; Castel and Martienssen, 2013). The important role of RNA silencing in antiviral defense has been well established in plants, insects, fungi, and mammals (Ding, 2010; Li et al., 2013). To counteract antiviral RNA silencing, most of the plant viruses encode silencing suppressor proteins (Li and Ding, 2006; Pumplin and Voinnet, 2013; Csorba et al., 2015).

In this review, we summarize the current information on the molecular aspects of antiviral RNA silencing in roots, with emphasis on the interactions between host antiviral defense and soil-borne viruses. Although the studies and information regarding this topic are still limited and mostly based on analyses using model plant-virus pathosystems, presently available information provides an insight into the divergent action of antiviral RNA silencing defense in roots relative to that already established for shoots. In addition, the effectivity of RNA silencing-based engineered resistance against soil-borne virus infection in plants is also briefly discussed.

## Diversities of Soil-Borne Viruses and Their Vectors

**FIGURE 1:**
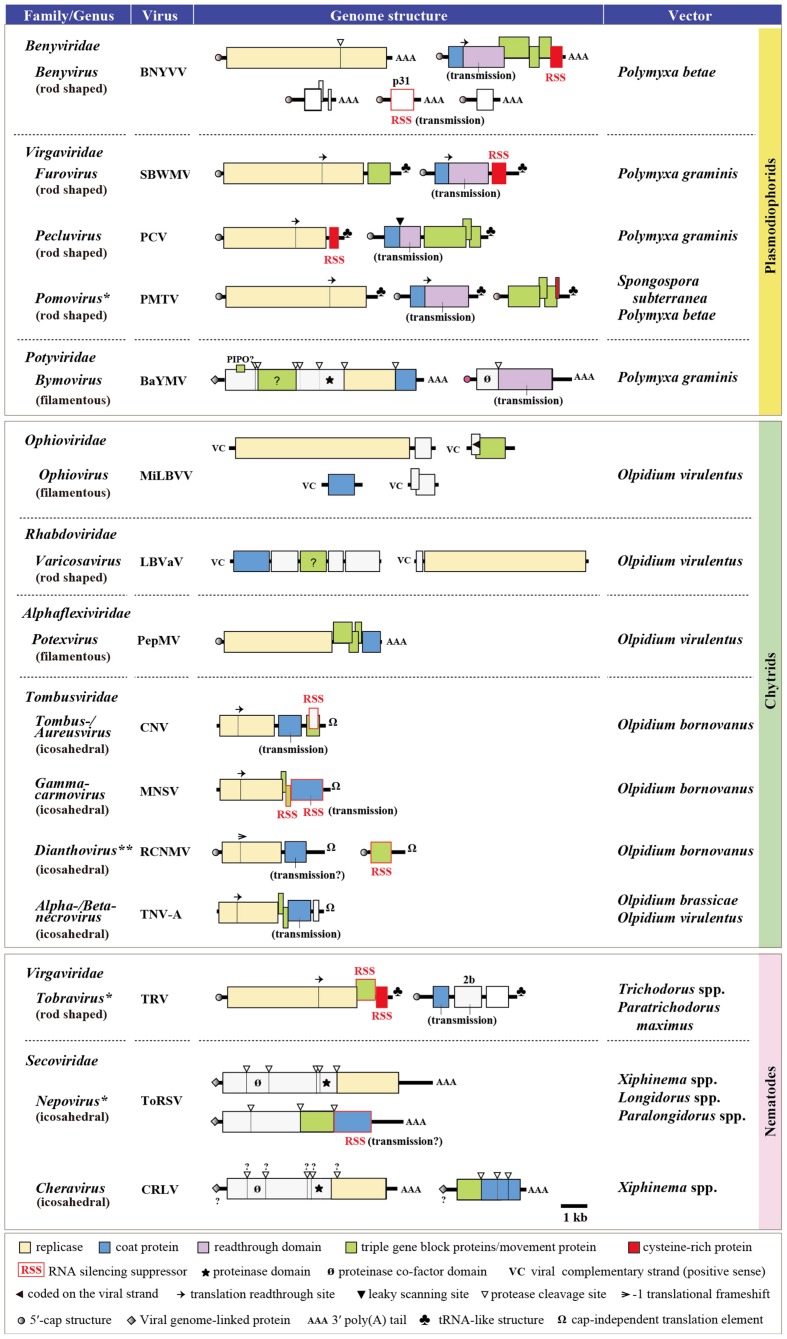
**Genome structure of the representative soil-borne plant viruses.** The type species member from each virus genus is presented except for the MiLBVV, PePMV, cucumber necrosis virus (CNV), melon necrotic spot virus (MNSV), and RCNMV, which are selected because they are transmitted by soil-borne vectors, while the vector of other members within the same genus is unknown and/or insects. ^∗^Some members of these genera are also known as seed transmissible. ^∗∗^A member of this genus (carnation ringspot virus) is transmitted by both *Longidorus* and *Xiphinema* spp. BNYVV, beet necrotic yellow vein virus; SBWMV, soil-borne wheat mosaic virus; PCV, peanut clump virus; PMTV, potato mop-top virus; BaYMV, barley yellow mosaic virus; MiLBVV, mirafiori lettuce big-vein virus; LBVaV, lettuce big-vein associated virus; PepMV, pepino mosaic virus; CNV, cucumber necrosis virus; MNSV, melon necrotic spot virus; RCNMV, red clover necrotic mosaic virus; TNV-A, tobacco necrosis virus-A; TRV, tobacco rattle virus; ToRSV, tomato ringspot virus; CRLV, cherry rasp leaf virus.

Currently, a number of plant single-stranded RNA (ssRNA) viruses belonging to at least 17 genera in eight virus families, but no DNA or dsRNA virus, are known to be transmitted by soil-inhabiting organisms (**Figure 1**). Considering the possible occurrence of non-vectored soil transmission of plant viruses (Campbell, 1996) and that the natural vectors of many plant viruses are still unknown, it is likely that the members of soil-borne viruses extend beyond these 17 genera. The vectors of soil-borne viruses could be largely categorized into three groups, namely, plasmodiophorids (a class within the kingdom Protista), *Olpidium* spp. (a genus of the order Chytridiales within the kingdom Fungi), and nematodes (a phylum within the kingdom Animalia) (**Figure 1**). *Olpidium* (*Olpidium virulentus*, *O. brassicae*, and *O. brassicae*) vectors transmit viruses from the families *Ophioviridae* (genus *Ophiovirus*), *Rhabdoviridae* [a previously free-floating genus *Varicosavirus*, but has recently been classified into this family (Afonso et al., 2016)], *Alphaflexiviridae* (genus *Potexvirus*), and *Tombusviridae* (genera *Tombus-*, *Aureus-*, *Gamma carmo*-, *Diantho*-, *Alphanecro*-, and *Betanecrovirus*), having flexuous, rod-shaped or icosahedral particles. Plasmodiophorids (*Polymyxa betae*, *P. graminis*, and *Spongospora subterranea*) are vectors of viruses from the families *Benyviridae* (genus *Benyvirus*), *Virgaviridae* (genera *Furo*-, *Peclu*-, and *Pomovirus*) and *Potyviridae* (genus *Bymovirus*), with rod-shaped or filamentous virions (except for two unclassified watercress viruses), while nematodes (*Longidorus* spp., *Paralongidorus maximus*, *Xiphinema* spp., *Trichodorus* spp., and *Paratrichodorus* spp.) are vectors of viruses from the families *Virgaviridae* (genus *Tobravirus*), *Secoviridae* (genus *Nepo-* and *Cheravirus*), and *Tombusviridae* (genus *Dianthovirus*), with rod-shaped or icosahedral particles. Thus, there is no specific association of each vector group with a particular structure of the viruses they transmit and likewise, the same vector species (f. e. *O. virulentus*) can transmit viruses with different particle structures. All known vector-transmitted soil-borne viruses have positive-sense ssRNA genomes except for the members of two genera, *Ophiovirus* and *Varicosavirus*, that have negative sense ssRNA genomes (Verchot-Lubicz, 2003; Kormelink et al., 2011) (**Figure 1**). It appears that the members with multipartite ssRNA genomes dominate the soil-borne viruses as they are more evident in the viruses that belong to the families *Rhabdoviridae* and *Potyviridae*, wherein the members having monopartite genomes and arthropod vectors (such as aphids, whiteflies, leaf- and planthoppers) are the majority in these virus families (Bragard et al., 2013). For soil-borne viruses with icosahedral virion, viral coat protein (CP) is apparently sufficient to mediate the transmission process, which is due to the direct attachment of the virus particles to the surface of vector zoospores or the retention of virions within the nematode feeding apparatus, while those with rod-shaped or filamentous virions involve additional specific proteins or protein domains located in CP read through proteins that facilitate the vector transmission, possibly either through forming a bridge between virus particles and vector or through other unknown mechanisms (Adams et al., 2001; Macfarlane, 2003; Bragard et al., 2013) (**Figure 1**).

*Olpidium* and nematode vectors transmit viruses to a wide range of hosts, particularly vegetable, ornamental and fruit plants, while viruses transmitted by plasmodiophorid vectors have a more limited range of hosts, but are important food crops such as cereals (furo- and bymoviruses), sugar beet and rice (benyviruses), peanut (pecluviruses), and potato (pomoviruses). For more details and comprehensive reviews regarding the vectors and genomes of soil-borne viruses, readers are referred to Brown et al. (1995), Rush (2003), Rochon et al. (2004), Kühne (2009), Bragard et al. (2013), Tamada and Kondo (2013), and Syller (2014) and references therein.

## Diseases Caused By Soil-Borne Viruses in Crops

Although soil-borne viruses enter the host plants via the roots, none of the members of this virus group is known to exhibit root tropism within the host plants. After initial infection in the roots, the soil-borne viruses usually travel long distances upward through vasculature and may subsequently induce various viral symptoms in the aerial plant part or may not generate any obvious symptoms, depending on the combination of virus and host plant. Only a few soil-borne viruses cause a particular disease symptom in roots or underground plant organs. Beet necrotic yellow vein virus (BNYVV; genus *Benyvirus*) infection in sugar beet causes the economically significant rhizomania disease which spreads worldwide (Tamada, 2016). It is typically characterized as a massive proliferation of lateral roots and rootlets (“bearded”-like appearance) and severely stunted taproots (Tamada, 1999). Potato mop-top virus (PMTV; genus *Pomovirus*) causes brown arcs or rings in potato tuber flesh (spraing symptoms; Harrison and Reavy, 2002). Viruses belonging to the genera *Furovirus* (type species *Soil-borne wheat mosaic virus*) and *Bymovirus* (type species *Barley yellow mosaic virus*) infect winter cereal crops and cause yellow mosaic symptoms on leaves as well as plant stunting (Kühne, 2009). Peanut clump virus (PCV; genus *Pecluvirus*) infection induces mottling and chlorotic ring symptoms on leaves as well as stunting of the plant (Thouvenel and Fauquet, 1981; Dieryck et al., 2009). The co-infection of lettuce big-vein associated virus (LBVaV; genus *Varicosavirus*) and Mirafiori lettuce big-vein virus (MiLBVV; genus *Ophiovirus*) is associated with lettuce big-vein disease in the field, which is characterized as mottling and chlorophyll clearing along the veins (appearing as big vein), but only MiLBVV is believed to be a sole disease agent (Maccarone, 2013). Viruses of the genera *Tombusvirus* (cucumber necrosis virus; CNV) and *Carmovirus* (i.e., melon necrotic spot virus, MNSV) cause necrosis or necrotic lesions on leaves and stems of Cucurbitaceae plants such as cucumber, melon, and squash (Dias and McKeen, 1972; Hibi and Furuki, 1985). Nepoviruses cause various diseases in a broad range of crops including fruit trees, vegetables, and ornamentals (Sanfaçon, 2008). Grapevine fanleaf virus (GFLV, genus *Nepovirus*) is the main causal agent of fanleaf and yellow mosaic diseases of grapevine worldwide (Andret-Link et al., 2004). Tobacco rattle virus (TRV, genus *Tobravirus*) can infect variety of crops and causes the major diseases of potato (spraing) and ornamental bulbs (Macfarlane, 2008).

## Genetic Components of Antiviral RNA Silencing in Plants

In plant, RNA silencing is initiated when imperfect or true double-stranded RNAs (dsRNAs) derived from cellular sequences or viral genomes, are processed by a ribonuclease III-like protein in the Dicer family called “Dicer-like (DCL) proteins” to generate 21–22-nucleotide (nt) microRNAs (miRNAs) or 21–26-nt short interfering RNA (siRNA) duplexes. Each strand of small RNA is then incorporated into the effector complexes termed “RNA-induced silencing complexes (RISCs),” which contain ARGONAUTE (AGO) proteins, to guide the sequence specificity in the downregulation processes (Axtell, 2013; Martínez de Alba et al., 2013; Bologna and Voinnet, 2014). Plant-encoded RNA-dependent RNA polymerases (RDRs) could contribute to the generation of dsRNA substrates for DCL processing, leading to either initiation of RNA silencing or production of secondary small RNAs that further intensify the potency of RNA silencing (Dalmay et al., 2000b; Wang et al., 2010). Plants encode multiple DCL, AGO, and RDR proteins to cope with diverse endogenous RNA-silencing pathways (Zhang et al., 2015). For example, the experimental model plant *Arabidopsis thaliana*, which is widely used for genetic studies on the RNA silencing mechanism, contains 4 DCL, 10 AGO, and 6 RDR proteins (Bologna and Voinnet, 2014). In *A. thaliana*, DCL4 and DCL2, which generate 21 and 22-nt siRNAs, respectively, act hierarchically in antiviral defense against RNA viruses. DCL4 is the primary DCL component for antiviral response, while DCL2 could functionally substitute DCL4 when it is overcome or absent (Deleris et al., 2006; Diaz-Pendon et al., 2007), but in some cases, DCL2 appears to have a specific role in the blocking of the systemic spread of viruses (Garcia-Ruiz et al., 2010; Andika et al., 2015a,b). Among 10 *A. thaliana* AGOs, AGO1 and AGO2 broadly function in antiviral defense against a wide range of RNA viruses, although other AGOs, such as AGO4, AGO5, AGO7, and AGO10, could also show antiviral activities in a more specific virus-host combination (Mallory and Vaucheret, 2010; Pumplin and Voinnet, 2013; Ma et al., 2014; Brosseau and Moffett, 2015; Carbonell and Carrington, 2015; Garcia-Ruiz et al., 2015). *A. thaliana* RDR6 and, to a lesser extent, RDR1, are required for antiviral defense against an RNA virus via amplification of viral siRNAs mechanism (Wang et al., 2010, 2011). In addition to DCL, AGO, and RDR core enzymes, other protein components in the RNA silencing pathway contribute to antiviral defense in *A. thaliana*, such as dsRNA-binding protein 4 (DRB4), a DCL4-interacting protein (Qu et al., 2008; Jakubiec et al., 2012), SUPPRESSOR OF GENE SILENCING 3 (SGS3), a coiled-coil protein (Mourrain et al., 2000; Rajamäki et al., 2014), and HUA ENHANCER 1 (HEN1) which methylates the 2′ hydroxy groups at the 3′-end termini of small RNAs to protect them from degradation (Boutet et al., 2003; Zhang et al., 2012). In *Nicotiana benthamiana* (wild tobacco), which is the most widely used experimental model host of plant RNA viruses, the antiviral activities of RNA silencing components, including the homologs of DCL4, AGO1, AGO2, and RDR6 were also demonstrated (Qu et al., 2005; Schwach et al., 2005; Scholthof et al., 2011; Andika et al., 2015b; Gursinsky et al., 2015; Fátyol et al., 2016).

## Distinct Characteristics of Transgene and Endogenous RNA Silencing in Roots

The occurrence and mechanism of RNA silencing in the root organ initially received relativity less attention from plant researchers. However, a growing number of studies have analyzed gene regulation, involving RNA silencing in roots, and revealed some unique characteristics of RNA silencing in roots relative to those observed in leaves or other aerial plant parts. First, lower RNA silencing activities were observed in roots than in leaves when post-transcriptional gene silencing in transgenic plants was induced by the sense transgene. In silenced transgenic *A. thaliana* lines carrying transgene encoding a Fab antibody fragment, suppression of the transgene expression was significantly lower in roots than in leaves (de Wilde et al., 2001). Co-suppression of tobacco endoplasmic reticulum ω-3 fatty acid desaturase (*NtFAD3*) gene by the sense transgene is effective in leaves but not in roots, although transgene-derived siRNAs accumulate in both tissues (Tomita et al., 2004). Likewise, lower levels of transgene silencing in roots than in leaves of silenced transgenic *N. benthamiana* lines carrying the CP read through gene of BNYVV or green fluorescent protein (GFP) gene were observed, as indicated by incomplete degradation of transgene mRNAs and lower levels of transgene siRNAs accumulation (Andika et al., 2005). Moreover, transgene DNA cytosine methylation levels at non-symmetrical CpNpN (N is A, T, or C) but not symmetrical CpG or CpNpG context were lower in roots than in leaves (Andika et al., 2006). Nevertheless, suppression of the target gene appears to be equally effective in mature leaves and roots if inverted repeat (IR) transgenes that are designed to express dsRNAs are used to induce the silencing (Fusaro et al., 2006; Marjanac et al., 2009). The sense- and IR-mediated silencing differ in the initiation step, where sense- but not IR-mediated silencing, requires conversion of ssRNAs into dsRNAs by the activities of RDR6 together with SGS3 and SDE3 (RNA helicase; Dalmay et al., 2000b, 2001; Mourrain et al., 2000; Béclin et al., 2002). It is therefore possible that in roots, either biosynthesis of dsRNA by RDR6 is less efficient or DCL protein(s) do not efficiently process RDR6-dependent dsRNA substrates for siRNA production. Transcriptomic analysis in *A. thaliana*, *N. benthamiana*, and rice showed that the mRNA expressions of RNA silencing core genes in leaves and roots are similar (Kapoor et al., 2008; Nakasugi et al., 2013). Thus, the reason for differential activities of sense transgene silencing between leaves and roots remains unclear.

Recent studies revealed that down-regulation of endogenous gene expressions in root could involve mobile (non-cell autonomous) small RNAs. During the development of *A. thaliana* roots, miR165a and miR166b produced in endodermis cells move to neighboring stele to mediate the suppression of *PHABULOSA* gene transcripts in a dose-dependent manner (Carlsbecker et al., 2010; Miyashima et al., 2011). Grafting experiments using *A. thaliana* plants demonstrated that siRNAs could be transported from shoots to roots and then induce RdDM of transgene promoter (Molnar et al., 2010; Melnyk et al., 2011). Moreover, a portion of endogenous small RNAs in roots are derived from shoots and associated with RdDM of a large number of genome loci, including transposable elements and endogenous genes (Molnar et al., 2010; Lewsey et al., 2016).

## Activities of Antiviral RNA Silencing in Roots

Some studies have detected the accumulation of siRNAs derived from various ssRNA viruses in the roots of infected plants including *N. benthamiana*, tomato, cucumber, and melon (Andika et al., 2005, 2013, 2015b; Herranz et al., 2015), demonstrating that viruses indeed induce antiviral RNA silencing responses in roots. BNYVV siRNA accumulation is lower in roots than in leaves of *N. benthamiana* and inversely related with RNA genome accumulation (Andika et al., 2005), suggesting that BNYVV may more effectively suppress RNA silencing in roots than in leaves (further discussed in the next section). Potato virus X (PVX, genus *Potexvirus*, natural vector unknown) siRNA accumulation is much lower in roots than in leaves, but this is likely due to the low level of PVX genome replication in roots (Andika et al., 2015b). Analyses using next generation sequencing indicated that siRNAs derived from PVX, Chinese wheat mosaic virus (CWMV, genus *Furovirus*), melon necrotic ringspot virus (MNSV, genus *Carmovirus*), and prunus necrotic ringspot virus (PNRSV, genus *Ilarvirus*, pollen and thrips transmission) are predominantly 21 nt in both leaves and roots (Andika et al., 2013, 2015b; Herranz et al., 2015), indicating that DCL4 is also the major DCL component for biosynthesis of viral siRNAs in roots. Notably, the proportions of MNSV and PNRSV sense siRNAs were higher than those of antisense siRNAs in roots, while the proportions of both strands were equal in leaves (Herranz et al., 2015). This suggests that in roots, DCL proteins preferentially target the sense strand genome of these viruses through cleaving of the secondary structures within viral RNA to generate sense siRNAs (Herranz et al., 2015), although it is generally thought that DCL mainly processes dsRNA replication intermediates formed during RNA virus replication (Ding, 2010). However, we cannot rule out other possibilities, including long-distance movement of sense siRNAs to roots and/or specific processing of viral subgenomic RNAs in roots.

**FIGURE 2:**
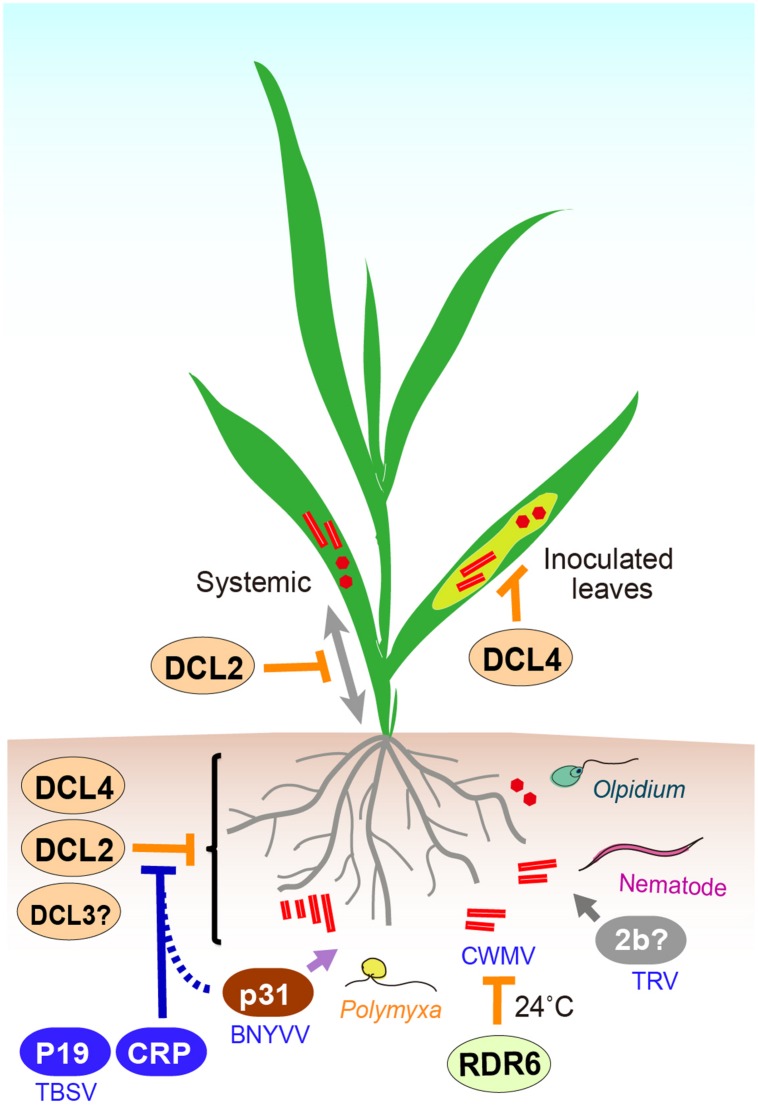
**A cartoon presentation illustrating the interplay between viruses and antiviral RNA silencing in roots.** In *Arabidopsis thaliana*, DCL4 is essential for the inhibition of PVX accumulation in inoculated leaves, while DCL2 particularly functions in blocking of PVX systemic infection. DCL4 is the primary DCL protein component involved in intracellular antiviral silencing in roots, but it can be functionally compensated for by DCL2 or possibly partially, DCL3. At higher temperatures (after a temperature shift to 24°C, see main text), RDR6 is involved in inhibition of CWMV multiplication in *Nicotiana benthamiana*, whereas at the same temperature other mechanism(s) is mainly responsible for CWMV inhibition in shoots. Cysteine rich proteins (CRPs) encoded by TRV and BNYVV more effectively suppress RNA silencing in roots than in leaves. BNYVV p31 exhibits root-specific RNA silencing suppression activity and contributes to efficient virus transmission by *Polymyxa betae* vector into roots. TBSV P19 expression is essential for TBSV infection via root mechanical inoculation in *N. benthamiana*.

Chinese wheat mosaic virus as well as other members of the genus *Furovirus* requires cool temperatures (below 20°C) to establish infection in the host plants (Ohsato et al., 2003). RDR6 is involved in temperature-dependent antiviral defense against RNA viruses in *N. benthamiana* leaves (Szittya et al., 2003; Qu et al., 2005). Knock-down of RDR6 homolog in *N. benthamiana* enables CWMV accumulation in roots but not in leaves, after a temperature shift to 24°C, and CWMV accumulation is associated with reduced accumulation of viral siRNAs in roots (Andika et al., 2013). This observation suggests that RDR6-dependent RNA silencing activity (probably through production of secondary siRNAs) is mainly responsible for inhibiting CWMV infection in roots at higher temperatures (**Figure 2**), whereas additional mechanism(s) are involved in the suppression of CWMV infection in leaves.

RNA silencing strongly inhibits PVX replication in roots of susceptible plants (Andika et al., 2012, 2015b). *A. thaliana* is not a susceptible host of PVX, but inactivation of DCL4 enables high accumulation of PVX in inoculated leaves, while inactivation of both DCL4 and DCL2 is required for systemic infection of PVX in upper leaves and roots. Another set of experiments was performed using a transgenic *A. thaliana* line that carries a replication-competent PVX cDNA transgene (AMP243 line; Dalmay et al., 2000a). Inactivation of DCL4 in AMP243 plants, where PVX replication is strongly suppressed in the cell due to intracellular antiviral silencing, is sufficient to enable high levels of PVX replication throughout the aerial organs, but not in roots (Andika et al., 2015b). These observations demonstrate that while DCL4 is critical for intracellular antiviral silencing against PVX replication in shoots, there are strong functional redundancies among DCL proteins, in which other DCLs (most probably DCL2) functionally complement DCL4 in roots (Andika et al., 2015a) (**Figure 2**). These strong redundancies may result in potent inhibition of PVX replication in roots, likely by providing multiple layers of antiviral defense. Thus, these observations suggest that to some degree, antiviral RNA silencing in roots may operate differently from that in shoots.

## Suppression of RNA Silencing By Soil-Borne Viruses

Numerous RNA silencing suppressors (RSSs) encoded by soil-borne viruses have been identified (listed in **Table 1**). Notably, the small cysteine-rich proteins (CRPs) located in a 3′proximal open reading frame (ORF) of the genome segment of viruses belonging to the genera *Beny-*, *Furo-*, *Peclu-*, and *Tobravirus* [as well as genera *Hordeivirus* (Yelina et al., 2002) and *Goravirus* (Atsumi et al., 2015) in the family *Virgaviridae*, some members are transmitted by seed transmission and no known biological vectors] (**Figure 1**), similarly function as an RSS, and some of them have been subjected to detailed studies. The CRP is also encoded by viruses belonging to the genus *Pomovirus*, but CRP encoded by PMTV does not exhibit RSS activity (Lukhovitskaya et al., 2005). The CRPs are characterized by the presence of multiple cysteine residues in their N-terminal or central portions, but they do not show a notable amino acid sequence similarity among different genera (Koonin et al., 1991). CRPs encoded by furo-, peclu- tobra-, and hordeiviruses contain a highly conserved CGxxH (Cys–Gly–x–x–His, x is any amino acid residue) motif (Te et al., 2005). Mutational analyses on CWMV 19K CRP and TRV 16K indicate that CGxxH motif as well as other conserved cysteine residues are critical for protein stability and/or RSS activity (Sun et al., 2013a; Fernández-Calvino et al., 2016). Similarly, cysteine residues located in a putative C4 (Cys4) zinc-finger domain of BNYVV p14, which are also conserved among other benyviruses, are essential for protein stability and RSS function (Chiba et al., 2013). Each of these CRPs shows distinct subcellular localization, for example BNYVV p14 localizes to nucleous (Chiba et al., 2013); CWMV 19K is associated with endoplasmic reticulum through amphipathic α-helix domain, and PCV P15 localizes to peroxisomes via C-terminal SKL (Ser-Lys-Leu) motif (Dunoyer et al., 2002; Sun et al., 2013a), although none of those organelle targeting is required for RSS activities. CWMV 19K and PCV P15 self-interact (dimerization) through coiled-coil domain (Dunoyer et al., 2002; Sun et al., 2013a), while the self interaction of BNYVV p14 is mediated by the C4 zinc-finger domain (Chiba et al., 2013) and importantly, the ability of those CRPs to form dimers is essential for RSS activities. CWMV 19K binds to the large form of CP (CUG-initiated extension to the N-terminal of CP), but the biological role of this interaction is unknown (Sun et al., 2013b). TRV 16K is not needed for the systemic spread of the virus, but is necessary for transient meristem invasion (Martín-Hernández and Baulcombe, 2008). In addition, TRV 16K inhibits the *de novo* formation of RISC and binds AGO4 (Fernández-Calvino et al., 2016), but does not cause a global deregulation of the microRNA-regulatory pathway (Martínez-Priego et al., 2008). Likewise, tomato ringspot virus (ToRSV, genus *Nepovirus*) CP binds and destabilizes AGO1 through the recognition involving WG/GW (Try-Gly/Gly-Try) motif (Karran and Sanfaçon, 2014). Nevertheless, the mechanism of action of other RSSs encoded by soil-borne viruses remains unclear.

**Table 1:** Properties of RNA silencing suppressors (RSSs) encoded by soil-borne viruses.

Genus Virus^1^	RSS	Protein category^2^	Local/cell-to-cell^3^	Motif, domain/target^4^	Subcellular localization	Di-mer	Reference
***Benyvirus***					
BNYVV	p14	CRP	Weak/-	NoLS, zinc-finger/-	Cytoplasm, nucleous	Yes	Andika et al., 2012; Chiba et al., 2013
	p31	-^7^	No/-		-	-	Rahim et al., 2007
BSBMV	p14	CRP	Weak/-	Zinc-finger/-	-	-	Chiba et al., 2013
BdMoV	p13	CRP	Weak/-	NLS, zinc-finger/-		-	Guilley et al., 2009; Andika et al., 2012
***Furovirus***					
SBWMV	19K	CRP	Weak/-	CGxxH, coiled-coil/-	-	-	Te et al., 2005
CWMV	19K	CRP	Weak/strong	CGxxH, coiled-coil, amphipathic α-helix/-	Endoplasmic reticulum	Yes	Sun et al., 2013a
***Pecluvirus***					
PCV	P15	CRP	Strong/-	CGxxH, coiled-coil, SKL/-	Peroxisomes	Yes	Dunoyer et al., 2002
***Tobravirus***					
TRV	16K	CRP	Weak/-	CGxxH/AGO4	Cytoplasm, nucleus	Yes	Ghazala et al., 2008; Andika et al., 2012; Fernández-Calvino et al., 2016
	29K^5^	MP	No/-				Deng et al., 2013
PepRSV	12K	CRP	Strong/-	-/-	-	-	Jaubert et al., 2011
***Tombusvirus***					
CNV	p20	(RSS)	Weak/-	-/-	-	-	Hao et al., 2011
***Gammacarmovirus***					
MNSV	p42	CP	Weak/strong	-/-	-	Yes	Genoves et al., 2006
	p7B	MP	Weak/-	-/-	-	-	Genoves et al., 2006
***Dianthovirus***					
RCNMV	RNA^6^		Strong/-	-/-	-	-	Takeda et al., 2005
	MP	MP	No/strong	-/-	Endoplasmic reticulum, cell wall	-	Tremblay et al., 2005; Powers et al., 2008; Kaido et al., 2009
***Nepovirus***					
ToRSV	CP	CP	Weak/-	WG/AGO1	-	Yes	Karran and Sanfaçon, 2014


*^1^BNYVV, beet necrotic yellow vein virus; BSBMV, beet soil-borne mosaic virus, BdMoV, burdock mottle virus; SBWMV, soil-borne wheat mosaic virus; CWMV, Chinese wheat mosaic virus; PCV, peanut clump virus; TRV, tobacco rattle virus; PepRSV, pepper ringspot virus; CNV, cucumber necrosis virus; TBSV, tomato bushy stunt virus; MNSV, melon necrotic spot virus; RCNMV, red clover necrotic mosaic virus, ToRSV, tomato ringspot virus.*

*^2^CRP, cysteine-rich protein; CP, coat protein; MP, movement protein.*

*^3^Suppression activities on local silencing in *Agrobacterium* co-infiltration assay relative to well-known strong suppressors such as p19 of tomato bushy stunt virus and HC-Pro of potato virus Y/ability to promote cell-to-cell movement of a suppressor-defective virus in the *trans*-complementation assay.*

*^4^NoLS, nucleolar-localization signal; NLS, nuclear-localization signal; CGxxH, cysteine-glycine-two any amino acid residues-histidine motif; SKL, serine-lysine-leucine motif; WG, tryptophan-glycine motif.*

*^5^Silencing suppression by 29K occurred in the context of RNA1 replication.*

*^6^RNA silencing suppression is mediated by the replication of RCNMV RNA1.*

*^7^“-”, not determined.*

It is important to point out that in *Agrobacterium* co-infiltration assay using a GFP reporter gene in *N. benthamiana* (Voinnet et al., 2000), a method that is most commonly used for identification of viral RSS, the majority of RSSs encoded by soil-borne viruses exhibit weak suppression activities against local silencing relative to suppression activities of well-known potent suppressors such as HC-Pro of potato virus Y (PVY, a potyvirus, aphid transmission) and p19 of tomato bushy stunt virus (TBSV, a tombusvirus, natural vector unknown; **Table 1**) (Verchot-Lubicz, 2003). However, some of those RSSs show strong activities to promote cell-to-cell movement of a suppressor-defective virus in *trans*-complementation assays (Genoves et al., 2006; Powers et al., 2008; Sun et al., 2013a), suggesting that those RSSs are more effective in inhibition of cell-to-cell spread of silencing signals that move ahead of the virus (intracellular silencing) rather than inhibition of local (intercellular) silencing in leaves. Interestingly, in a silencing reversal assay using transgenic *N. benthamiana* line 16c systemically silenced for the GFP gene, BNYVV or TRV infection restored GFP expression in roots but not in leaves, while infection of tobacco mosaic virus (TMV, genus *Tobamovirus*) and two aphid-borne (non-soil-borne) viruses, PVY and cucumber mosaic virus (genus *Cucumovirus*), restored GFP expression in both tissues. Moreover, BNYVV and TRV elevated PVX RNA accumulation in a co-infection experiment and this stimulating effect was due to the activity of p14 or 16K RSS encoded by those viruses (Andika et al., 2012). In another co-infection experiment, TRV also showed an activity to suppress antiviral silencing-like responses that inhibit the replication of TMV in lateral root primordia (Valentine et al., 2002). Collectively, these observations suggest that some RSS encoded by soil-borne viruses might be more effective in roots than in leaves. Further supporting evidence for this notion comes from the analyses of accumulations of some soil-borne viruses in plants. CWMV and MNSV accumulate to higher levels in roots than in leaves (Gosalvez-Bernal et al., 2008; Andika et al., 2013). Nepo- or tobraviruses have unusual ability to infect meristems and often show a recovery phenotype, which is manifested as a drastic reduction in virus symptoms and titer in newly developed leaves (Ratcliff et al., 1997, 1999). The recovery phenotype is thought to be mediated by RNA silencing-related mechanisms and mutations in the viral RSS can result in viruses that exhibit a recovery-like phenotype in the host plants (Ratcliff et al., 1997; Szittya et al., 2002). Similarly, BNYVV infection in *N. benthamiana* exhibited reduced viral accumulation similar as the “recovery” phenomenon in leaves but not in roots (Andika et al., 2005). Therefore, it is suggested that the weak RSS encoded by these viruses could not effectively inhibit the induction of antiviral systemic silencing, leading to recovery in upper leaves (Martín-Hernández and Baulcombe, 2008; Ghoshal and Sanfaçon, 2015).

Only a few studies have examined the relevance of silencing suppression in the context of virus infection through roots. The p31 encoded by RNA 4 of BNYVV is not essential for virus multiplication, but is required for efficient virus transmission by *P. betae* vector into roots (Tamada et al., 1989). Interestingly, in a silencing reversal assay, p31 showed an activity to suppress GFP transgene silencing in roots but not in leaves, proving that p31 has a root-specific RSS function (Rahim et al., 2007) (**Figure 2**). TRV 2b is a nematode transmission helper protein (Macfarlane, 2003) and is also required for extensive root (and also shoot) meristem invasion (Valentine et al., 2004). In a more recent study on TBSV, which is also considered a soil-borne virus because soil solarization and fumigation could reduce disease incidence (Gerik et al., 1990; Campbell, 1996), TBSV p19 suppressor is required for TBSV to infect *N. benthamiana* via root mechanical inoculation but not via leaves mechanical inoculation (Manabayeva et al., 2013) (**Figure 2**). Together, these observations suggest that suppression of RNA silencing or other antiviral defense mechanism is one of the factors that determine the efficiency of virus transmission to the roots.

## Effectivity of RNA-Based Transgenic Resistance Against Soil-Borne Viruses

Using the transgenic approach, RNA silencing has been successfully applied to generate plant resistant against infection with diverse viruses (Simon-Mateo and Garcia, 2011; Cillo and Palukaitis, 2014; Saurabh et al., 2014). Several researches have introduced a portion of genome sequence derived from soil-borne viruses into either experimental models or crops plants and evaluated the responses of the transgenic plants to virus infection. Although the silencing of viral sequences in the transgenic plants could in general provide a high degree of protection against the soil-borne viruses (e.g., for crops, Dong et al., 2002; Pavli et al., 2010; Zare et al., 2015; Kawazu et al., 2016), some other studies similarly observed that upon roots inoculation, virus resistance was less effective in roots than in shoots. Inoculation of roots of transgenic *N. benthamiana* carrying CP gene of PMTV using viruliferous *S. subterranea* resulted in virus accumulation in roots but no systemic movement of PMTV to shoots (Germundsson et al., 2002). *N. benthamiana* plants transformed with CP read through domain of BNYVV were immune to viral infection following leaf mechanical infection, but BNYVV accumulated at a low level in the roots of the same plants upon challenged by viruliferous *P. betae* vector (Andika et al., 2005). Transgenic *N. tabacum* carrying 57-kDa read through domain of the replicase gene of TRV was highly resistant to manual leaf inoculation, but the virus could be detected in roots following root manual inoculation or nematode vector inoculation (Vassilakos et al., 2008). Likewise, MiLBVV was detected in roots, but not in leaves of transgenic lettuce carrying IR transgene of MiLBVV CP following roots inoculation by *Olpidium* vectors (Kawazu et al., 2009). However, transgenic sugar beet plants carrying 0.4 kb IR sequence of BNYVV replicase gene showed high resistance to BNYYV infection through vector inoculation (Lennefors et al., 2008). This suggests that the potency of transgenic resistance against root inoculation could be affected by various factors including construct design, viral gene sequence, and plant species. A recent report showed that a high and durable transgenic wheat resistance against wheat yellow mosaic virus (WYMV, genus *Bymovirus*) infection in the field is obtained by transformation with antisense nuclear inclusion b (NIb) replicase of WYMV (Chen et al., 2014). Transgene siRNAs are not detected in transgenic plants, indicating that the resistance is not mediated by transgene silencing, although it is possible that the resistance resulted from cleavage of dsRNAs that are formed through annealing of antisense transcripts with viral genome RNA by DCLs or other cellular RNases (Chen et al., 2014). It is necessary to further explore the antiviral efficacy of antisense transgenes in different soil-borne virus-host plant pathosystems. In addition, the effectivity of artificial miRNAs in conferring virus resistances (Niu et al., 2006; Qu et al., 2012; Ramesh et al., 2014) has not been tested against soil-borne viruses.

## Concluding Remarks

Overall, the observations from the studies described in this review provide evidence for divergent operations of RNA silencing in roots, although the primary factors responsible for the distinct regulation of RNA silencing activities in roots remain an open question. Moreover, the antiviral roles of RNA silencing components in the context of virus infection through roots are yet to be examined. Interestingly, those studies also demonstrated that some soil-borne viruses appear to have adapted to the mechanistic differences of antiviral RNA silencing in roots by evolving their RSS with more active function in facilitating viral transmission and accumulation in roots than in leaves. Further studies are needed to investigate the possibility that RSS encoded by soil-borne viruses specifically target certain molecular components of antiviral silencing in roots. It is worth mentioning that many plant viruses vectored by sap-sucking insects that usually penetrate their stylets into the phloem tissue, exhibit phloem-limited accumulation within their host plants (Omura et al., 1980; Latham et al., 1997; de Zoeten and Skaf, 2001; Shen et al., 2016). This also goes along with the opinion that the vectors influence virus evolution and adaptation within the host plants.

An agronomic practice for the effective control of soil-borne diseases is not available, while the use of methyl bromide (bromomethane), which is the most popular pre-plant soil fumigant against soil-borne fungi and nematodes, has been withdrawn worldwide under the Montreal protocol (Bell, 2000). Thus, harnessing the plant natural antiviral defense could potentially become a feasible alternative method for protecting the crop plants against these diseases. In fact, the results of several studies have opened the possibility of RNA silencing enhancement in plants, for example by chemical (ascorbic acid derivatives) treatments (Fujiwara et al., 2013), environmental (light intensity and temperature) modifications (Kotakis et al., 2010; Patil and Fauquet, 2015), overexpression of endogenous plant RNA silencing enhancers (Dorokhov et al., 2006; Meyer et al., 2015) and deactivation of plant endogenous suppressor of RNA silencing (Sarmiento et al., 2006; Gy et al., 2007; Shamandi et al., 2015; Liu and Chen, 2016). With the notion that RNA silencing plays an important role in defense against virus invasion via roots, it is anticipated that more detailed studies on antiviral RNA silencing mechanisms in roots could provide a solid basis for the future development of effective control measures of soil-borne virus diseases. Lastly, the advent of novel molecular tools for functional genomics and expanding understanding of plant innate immunity will allow greater options for the development of virus resistant crops.

## Author Contributions

All authors listed, have made substantial, direct and intellectual contribution to the work, and approved it for publication.

## Conflict of Interest Statement

The authors declare that the research was conducted in the absence of any commercial or financial relationships that could be construed as a potential conflict of interest.
